# Management of Thyrotoxicosis in Children and Adolescents: A Turkish Multi-center Experience

**DOI:** 10.4274/jcrpe.galenos.2018.2018.0210

**Published:** 2019-05-28

**Authors:** İhsan Esen, Elvan Bayramoğlu, Melek Yıldız, Murat Aydın, Esin Karakılıç Özturhan, Zehra Aycan, Semih Bolu, Hasan Önal, Yılmaz Kör, Deniz Ökdemir, Edip Ünal, Aşan Önder, Olcay Evliyaoğlu, Atilla Çayır, Mehmet Taştan, Ayşegül Yüksel, Aylin Kılınç, Muammer Büyükinan, Bahar Özcabı, Onur Akın, Çiğdem Binay, Suna Kılınç, Ruken Yıldırım, Emel Hatun Aytaç, Elif Sağsak

**Affiliations:** 1 Fırat University Faculty of Medicine, Department of Pediatric Endocrinology, Elazığ, Turkey; 2 Dr. Sami Ulus Maternity and Children’s Disease Training and Research Hospital, Clinic of Pediatric Endocrinology, Ankara, Turkey; 3 Kanuni Sultan Süleyman Training and Research Hospital, Clinic of Pediatric Endocrinology, İstanbul, Turkey; 4 Ondokuz Mayıs University Faculty of Medicine, Department of Pediatric Endocrinology, Samsun, Turkey; 5 İstanbul University İstanbul Faculty of Medicine, Department of Pediatric Endocrinology, İstanbul, Turkey; 6 Düzce University Faculty of Medicine, Department of Pediatric Endocrinology, Düzce, Turkey; 7 Adana City Hospital, Clinic of Pediatric Endocrinology, Adana, Turkey; 8 Dicle University Faculty of Medicine, Department of Pediatric Endocrinology, Diyarbakır, Turkey; 9 Göztepe Training and Research Hospital, Clinic of Pediatric Endocrinology, İstanbul, Turkey; 10 İstanbul University Cerrahpaşa Faculty of Medicine, Department of Pediatric Endocrinology, İstanbul, Turkey; 11 Erzurum Bölge Training and Research Hospital, Clinic of Pediatric Endocrinology, Erzurum, Turkey; 12 Çukurova University Faculty of Medicine, Department of Pediatric Endocrinology, Adana, Turkey; 13 Derince Training and Research Hospital, Clinic of Pediatric Endocrinology, Kocaeli, Turkey; 14 Gazi University Faculty of Medicine, Department of Pediatric Endocrinology, Ankara, Turkey; 15 Konya Training and Research Hospital, Clinic of Pediatric Endocrinology, Konya, Turkey; 16 Zeynep Kamil Maternity and Children’s Disease Training and Research Hospital, Clinic of Pediatric Endocrinology, İstanbul, Turkey; 17 Gülhane Training and Research Hospital, Clinic of Pediatric Endocrinology, Ankara, Turkey; 18 Çorlu State Hospital, Clinic of Pediatric Endocrinology, Tekirdağ, Turkey; 19 Bağcılar Training and Research Hospital, Clinic of Pediatric Endocrinology, İstanbul, Turkey; 20 Diyarbakır Pediatric Hospital, Clinic of Pediatric Endocrinology, Diyarbakır, Turkey; 21 Gaziantep University Faculty of Medicine, Department of Pediatric Endocrinology, Gaziantep, Turkey; 22 Gaziosmanpaşa Taksim Training and Research Hospital, Clinic of Pediatric Endocrinology, İstanbul, Turkey

**Keywords:** Graves’ disease, hashitoxicosis, thyrotoxicosis, antithyroid drug, radioactive iodine, total thyroidectomy

## Abstract

**Objective:**

To determine the demographic and biochemical features of childhood and juvenile thyrotoxicosis and treatment outcome.

**Methods:**

We reviewed the records of children from 22 centers in Turkey who were diagnosed with thyrotoxicosis between 2007 to 2017.

**Results:**

A total of 503 children had been diagnosed with thyrotoxicosis at the centers during the study period. Of these, 375 (74.6%) had been diagnosed with Graves’ disease (GD), 75 (14.9%) with hashitoxicosis and 53 (10.5%) with other less common causes of thyrotoxicosis. The most common presenting features in children with GD or hashitoxicosis were tachycardia and/or palpitations, weight loss and excessive sweating. The cumulative remission rate was 17.6% in 370 patients with GD who had received anti-thyroid drugs (ATDs) for initial treatment. The median (range) treatment period was 22.8 (0.3-127) months. No variables predictive of achieving remission were identified. Twenty-seven received second-line treatment because of poor disease control and/or adverse events associated with ATDs. Total thyroidectomy was performed in 17 patients with no recurrence of thyrotoxicosis and all became hypothyroid. Ten patients received radioiodine and six became hypothyroid, one remained hyperthyroid and restarted ATDs and one patient achieved remission. Two patients were lost to follow up.

**Conclusion:**

This study has demonstrated that using ATDs is the generally accepted first-line approach and there seems to be low remission rate with ATDs in pediatric GD patients in Turkey.

**What is already known on this topic?**

Graves’ disease is the most common cause of thyrotoxicosis in children and adolescents, as in adults. Management of thyrotoxicosis in children and adolescents is controversial and often unsatisfactory. There are no published data on clinical features and treatment outcomes of Turkish children and adolescents with thyrotoxicosis.

**What this study adds?**

Using anti-thyroid drugs (ATDs) with the hope that the patients will enter a remission over time is the generally accepted first-line approach in Turkey. This study shows that this approach achieved low remission rates, a result which was consistent with previous studies. Surgery is preferred to radioactive iodine ablation for patients with poor disease control and those experiencing adverse events associated with ATDs in Turkish children and adolescents with Graves’ disease.

## Introduction

Thyrotoxicosis is characterized by excess circulating thyroid hormones, irrespective of the source (1). Juvenile thyrotoxicosis can have various clinical manifestations, including adverse effects on growth and development and may cause pronounced neuropsychological manifestations (2). Graves’ disease (GD) is the most common cause of thyrotoxicosis in childhood (2). Incidence of GD increases with age, but is less common in children than in adults. An increase in the incidence rate of childhood GD has been reported by several studies (3,4,5,6).

Although a few treatment guidelines have been published, management of juvenile thyrotoxicosis is controversial and often unsatisfactory (7,8,9). Treatment options include anti-thyroid drugs (ATDs), radioactive iodine therapy (RAI) and surgical interventions. Most patients diagnosed with thyrotoxicosis are initially treated with ATDs, as there is a chance of remission with ATD therapy, although optimal treatment duration is controversial. Patients with thyrotoxicosis who do not respond to medical therapy or who have adverse reactions to ATDs must be managed with a second line treatment, such as RI or thyroid surgery. However, all of the treatment options have distinct advantages and disadvantages (10). There are novel approaches such as using rituximab, thyroid stimulating hormone (TSH) receptor (TSHR) specific peptides or monoclonal TSHR-blocking antibodies with the aim of ameliorating the immune dysfunction seen in GD. At present, there are very few ongoing studies in this area and some completed studies have shown conflicting results (11,12,13).

Remission has been reported in approximately one third of children with GD and relapse occured in half of the patients after remission was achieved (7,14,15,16,17,18,19,20,21). Remission rates are lower and relapse rates are higher in children than in adults (22). However, the definitions of remission and relapse also differed among studies.

In this study, we aimed to assess the demographic and biochemical features of children and adolescents with thyrotoxicosis, the preference of physicians for treatment options in juvenile thyrotoxicosis and management outcome in these patients.

## Methods

In December 2017, an online invitation was sent to all pediatric endocrinology departments across Turkey asking them, if they were willing, to review their patients under the age of 18 years who presented between 2007 and 2017 with elevated free thyroxine (fT4) concentrations, above the upper limit of the local reference range. Thus, the clinical and biochemical features, treatment preferences and outcome in relation to treatment were documented by analysis of these patient records returned by 22 pediatric endocrinology departments responding positively to the study invitation. Thyrotoxicosis was defined as an elevated fT4 and/or free tri-iodothyronine (fT3) concentration, above the upper limit of the local reference range, together with suppressed TSH levels, below the lower limit of the local reference range.

Data on the clinical features of the sample at first presentation included gender, age, clinical symptoms and anthropometric measurements which consisted of weight in kilograms to the nearest 0.1 kg, height in centimetres to the nearest 1 mm and body mass index (BMI) calculated by the formula weight in kilograms divided by the square of height in meters. Standard deviation scores (SDS) of weight, height and BMI of patients were calculated by using reference values for Turkish children (23).

Biochemical data collected included: serum alanine transaminase (ALT); aspartate transaminase (AST); TSH; fT4; fT3; anti-thyroid peroxidase (anti-TPO) antibodies; anti-thyroglobulin (anti-Tg) antibodies and anti-TSH receptor antibodies (TRAb). Commercial kits were used by the participating centers to assay these hormones and antibodies. Because different commercial kits had been used to assay TRAb among clinics and sometimes in the same clinic, TRAb/upper limit for TRAb ratio (TRAb ratio) was used in the data analysis and a TRAb ratio >1.0 was accepted as an elevated TRAb.

GD was defined as thyrotoxicosis with either elevated TRAb or clinical signs or findings suggestive of GD such as thyroid ophthalmopathy or diffuse uptake of radioisotope on thyroid scan or persistent thyrotoxicosis of more than two years standing without any other cause. Presence of ophthalmopathy was based on clinical reports of physicians. The thyrotoxic phase of chronic lymphocytic thyroiditis (hashitoxicosis) was defined as thyrotoxicosis with the presence of at least one of the anti-TPO or anti-Tg antibodies (based on the reference range of locally used commercial kits) in patients without any other cause.

The preferred approach to ATD treatment (block-and-replace or dose reduction), type of ATD [methimazole (MMI) or propylthiouracil (PTU)], duration of therapy, side effects and date/age of stopping treatment and if the patient subsequently relapsed and underwent RAI treatment or surgery were also recorded. Remission was defined as biochemical euthyroidism at the time of collecting data after cessation of ATD for at least three months.

Patients were classified by their final diagnosis and the results were expressed in percentages. Patients with GD or hashitoxicosis have been analyzed in greater detail. Insufficient data, a diagnosis of subclinical hyperthyroidism (low serum TSH, but normal fT4 and fT3 concentration) and gestational thyrotoxicosis were considered as exclusion criteria.

Ethical approval for this study was obtained from Ethical Committee of the Fırat University Medical School (05.10.2017-0015). Informed consent was not obtained from patient’s parents because this paper does not report on experimental protocol and all data analyzed were collected as part of routine diagnosis and treatment options.

### Statistical Analysis

Continuous variables were described as medians and ranges or means ± SD. Intergroup comparisons were performed using the Mann-Whitney U or Student’s t-test. χ^2^ test was used for categorical variables. Multiple regression analysis was used to determine whether age, sex, weight SDS, height SDS, BMI SDS, fT4, fT3, TRAb ratio at diagnosis and duration of ATD had independent associations with occurring relapse in the patients’ group in which ATD therapy was stopped for possible remission. Statistical significance was assumed if p<0.05 and all analyses were performed using IBM SPSS Statistical Software (version 22, SPSS Inc., Chicago, IL, USA).

## Results

Case records of 514 patients were received and 11 patients were excluded due to: insufficient data (n=2); duplication (n=4); diagnosis of subclinical hyperthyroidism (n=4); or gestational thyrotoxicosis (n=1). Thus, between 2007 and 2017, the medical records of 503 eligible children from 22 institutions in 12 different cities were reviewed. Of the 503 patients who were included in the study, 375 (74.6%) were diagnosed with GD and 75 (14.9%) patients had hashitoxicosis. The diagnosis in the remaining patients were thyroid hormone receptor-beta mutation in 22 (4.4%), subacute thyroiditis in four (0.8%), toxic nodular goiter in four (0.8%), neonatal GD in three (0.6%), papillary thyroid carcinoma in two (0.4%) and 18 patients (3.6%) who were not assigned a specific diagnosis. Diagnosis of patients with GD was mostly based on positive results for TRAb (81.6%) or elevated radioactive iodine or technetium (99mTc) uptake or observed ophthalmopathy (9.6%). Only 32 patients (8.8%) who were negative for TRAb or had no TRAb measurement had been assigned a diagnosis of GD based on clinical findings during follow up.

The most common reported presenting complaints among patients with GD or hashitoxicosis were tachycardia and/or palpitations, weight loss and excessive sweating. Distribution and frequencies of complaints at presentation were similar between groups, except that goiter and ocular symptoms were more frequent in patients with GD (see Table 1).

Clinical features of GD and hashitoxicosis are shown in Table 2. There was no significant difference between the two groups with regard to gender distribution, age at diagnosis, weight SDS, height SDS and BMI SDS. Patients with GD had higher median fT4 and fT3 levels. The mean starting dose of ATDs was significantly higher and duration of ATD therapy was significantly longer in patients with GD (p<0.05). With respect to medical treatment options, the vast majority of the patients were treated with MMI as ATD (89.2% and 91.3% for GD and hashitoxicosis, respectively) and subjected to a dose reduction regimen. The mean starting dose of ATDs was significantly higher in the GD than hashitoxicosis groups (Table 2).

**Figure 1:**
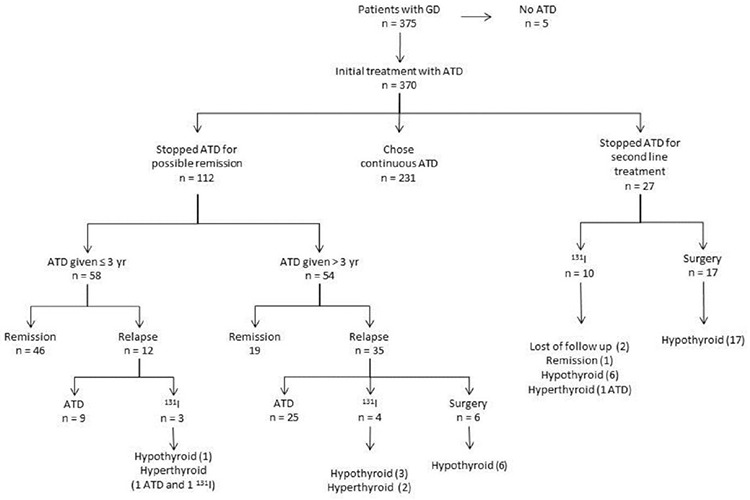
Outcome in 375 Turkish children and adolescents with Graves’ disease followed between 2007-2017 ATD: anti-thyroid drugs, GD: Graves’ disease

Outcome of patients with GD is shown in Figure 1.

Five of 375 (1.3%) patients with GD did not receive ATDs. These patients were managed only with beta-blockers. Approximately 62% (231/370) of patients who received ATDs initially were continued on ATD at the time of the last contact for data collection. The median (range) duration of ATD therapy in these patients was 16.1 (0.3-99.6) months. ATDs were stopped in approximately one-third of patients (112/370; 30.2%) for possible remission. In 58 patients, the trial of cessation of ATD was initiated in the first three years of treatment and 46 of these patients (79.3%) remained in remission. In 54 patients, requirement of ATD persisted and a trial of cessation of ATD attempted beyond three years of treatment. Of this group, 19 patients (35.2%) remained in remission. Forty-seven of 112 patients who relapsed after stopping ATD therapy for possible remission. Of these 34 remained on ATD, six underwent total thyroidectomy and all became hypothyroid. Seven of this group received radioiodine, four developing hypothyroidism and three remaining hyperthyroid. Two of three who remained hyperthyroid remained on ATD and the other received a second dose of radioiodine and thereafter became hypothyroid (Figure 1). The median (range) interval between ATD treatment cessation and relapse of hyperthyroidism was 6.0 (0.7-60.8) months. The cumulative remission rate was 17.6% in 370 patients with GD who received ATDs for initial treatment and they were treated for a median (range) of 22.8 (0.3-127) months. Clinical and biochemical features of patients with GD who stopped ATDs for remission or relapsed afterwards are shown in detail in Table 2. Patients who did not achieve remission had a lower BMI SDS at diagnosis, higher initial fT4 and fT3 concentrations and longer duration of ATD therapy (Table 3). However, these variables were not identified as independent predictors of relapse by regression analysis (Table 4). Four patients who had high AST and/or ALT levels did not remain euthyroid and relapsed after discontinuation of ATD.

Twenty-seven patients with GD (7.3%) received second-line treatment (surgery or radioactive iodine ablation) for poor disease control and adverse events associated with ATD (Figure 1). Total thyroidectomy was performed in 17 patients with no recurrence and all these patients became hypothyroid without severe complications. Ten patients received radioiodine; six became hypothyroid, one remained hyperthyroid and started taking ATD again, one achieved remission and outcome is unknown in two patients due to loss of follow up. There was no significant difference in the age of patients at second-line treatment time between patients who received radioiodine [16.1±2.8 years (range 10.8-19.4)] and patients who underwent surgery [15.2±2.4 years (range 9.3-19.6)].

Six of 75 patients with hashitoxicosis did not receive ATDs and were managed solely with beta-blockers. ATDs were stopped after a mean period of 9.3±6.3 months (range 0.7-22.5) in 32 patients with hashitoxicosis for possible remission and all of them achieved remission. The remaining 37 patients were continuing to use ATDs at their last visits with a mean duration of treatment of 8.0±6.9 (range 0.3-34.0) months.

## Discussion

This report is possibly one based on the second largest group of patients with childhood and juvenile thyrotoxicosis to date in the literature.

GD is the most common cause of the thyrotoxicosis in children and adolescents accounting for more than 95% of cases (2). However, the occurrence of GD was much lower in our series, accounting for approximately three quarters of all cases. The second most frequent cause of juvenile thyrotoxicosis is hashitoxicosis and its prevalence was reported to range from 0.5% to 22% in different studies (9). Our results were similar to those reported in a recent study from Scotland in which 19.6% of patients with thyrotoxicosis were classified as hashitoxicosis (20). Due to the similarity in the most commonly encountered presenting complaints in patients with GD and hashitoxicosis, the distinction between the two may be difficult, as was observed in this study. Additionally, most patients with hashitoxicosis may not have been diagnosed, probably because of its relatively short thyrotoxic course. The hallmark of GD is the presence of TRAb while patients with hashitoxicosis will typically have anti-TPO and/or anti-Tg (24,25). In the present study, most of the patients with GD were tested for the presence of TRAb and we believe this is one of the strong features of this study. On the other hand, TSHR antibodies may also be present in the sera of patients with hashitoxicosis and some experts consider GD and hashitoxicosis to be basically the same disorder at different ends of a continuum (26).

Graves’ ophthalmopathy is an inflammatory disease of the eye and orbital tissues, and its prevalence has been previously reported to range from 17.1-67.6% in children and adolescents with GD (21,27). A relatively high prevalence (86.8%) of eye signs in children with GD was reported in a recent study (20). This finding may be due to inclusion of mild signs such as lid lad in the analysis rather than assessing true proptosis. In the present study, ophthalmopathy was reported in approximately in one third of patients with GD and this finding is in concordance with previous studies. However our data on ocular findings were not based on a standard protocol for definition of Graves’ ophthalmopathy, but we suppose that reported cases presumably are children with moderate or severe ophthalmopathy.

Treatment options for GD in children include ATDs, RAI therapy and surgical thyroidectomy, and each of these treatment approaches is associated with specific risks (2). ATDs are generally the accepted option for initial treatment of GD in children and adolescents in most countries. In this study, almost all patients with GD received ATDs, with most being prescribed MMI, with the exception of a few patients who had been treated with beta-blockers. PTU is not recommended for use in children because of its potential severe hepatotoxicity (28). Thus, there was no patient who received PTU as initial treatment in this patient group. Despite the presence of various definitions of remission and a wide variety of ATD treatment durations from two years and beyond, remission rates after ATD withdrawal are reported to vary from 11% to 49% (7,19,21,22,29). In the present study, remission was achieved in 58.0% of patients at the time when their anti-thyroid treatment was ceased for possible remission. This relative high remission rate should be interpreted with caution due to the retrospective nature of this study and to the fact that there was no standard protocol applied to all patients in this study. The cumulative remission rate was 17.6% when remission rate in all patients who received ATDs for initial treatment was estimated, which is consistent with the literature.

In several studies, a range of prognostic factors have been identified as being associated with lower remission chance in children and adolescents with GD (22,30). These studies have reported that ethnicity, age, pubertal status, BMI (SDS), goiter volume, initial severity (higher fT4 concentration and TRAb levels) and presence of other autoimmune conditions are prognostic factors (14,15,16,31). However, similar to a number of other studies (6,7,17,32), no clinical variable that is constantly associated with a definite outcome was identified in our cohort. The major limitation of these studies was that almost all, including this one, are retrospective. Kaguelidou et al (16) have published the only prospective study which reported that the risk of relapse was higher in very young patients, in patients of non-Caucasian origin and those with high levels of serum TRAb and fT4. In our study, a higher remission rate in patients who received ATDs for less than three years indicates that patients who will achieve remission may be predicted, based on follow up of thyroid function tests and requirement of lower doses of ATDs. Thus, a trial of cessation of ATDs can be tried at an earlier time in such patients.

If remission is not achieved or relapse or severe side-effects occur during ATDs therapy, radioiodine ablation and surgical thyroidectomy are available treatment modes for second line approach in patients with GD. Thyroidectomy is an effective treatment for GD (8). In this study we found that this method had been used slightly more frequently than radioiodine ablation. Various studies reported that hypothyroidism occurs in nearly all children who undergo total thyroidectomy (20,21,33,34). Our observations were concordant with these studies.

In the past, 131I therapy was considered to be contraindicated in children. However, according guidelines from Japan and the American Thyroid Association, 131I therapy can be performed with caution (8,9). Due to the ease of administration of RAI there is a trend towards permanent therapy (10). Remission rates vary due to variability in the dose of 131I used. While many patients are successfully treated with 131I therapy, in approximately one third of patients remission is not achieved or relapse occurs (30). According to our data, 11 of the 17 children who were treated with 131I achieved biochemical remission. Fears about radioiodine ablation, compounded by the lack of access to RAI may have contributed to the relative low rate of 131I therapy in children with GD in this series.

### Study Limitations

Our study has potential limitations and strengths. The major limitation of this study is its retrospective nature and the lack of a globally accepted standard protocol for management of thyrotoxicosis in children and adolescents. In addition, adverse events associated with ATD treatment, thyroidectomy or radioactive iodine ablation were not included in the data collected for this study. The lack of detailed information about side effects hampered interpretation of the adverse events and treatment preferences. The strengths of this study are inclusion of data from multiple centers throughout Turkey and its relatively large sample size when compared with previous studies.

## Conclusion

In conclusion, clinical manifestations and laboratory findings of patients with GD and hashitoxicosis were found to be essentially similar. A positive TRAb result, elevated radioactive iodine (or 99mTc) uptake and ophthalmopathy are the key features for diagnosis of GD. Although there is no optimal accepted treatment for GD, we have observed that initial treatment with ATDs and using total thyroidectomy and 131I therapy as a second line treatment for permanent therapy is a generally accepted approach for treatment of patients with GD.

## Figures and Tables

**Table 1:**
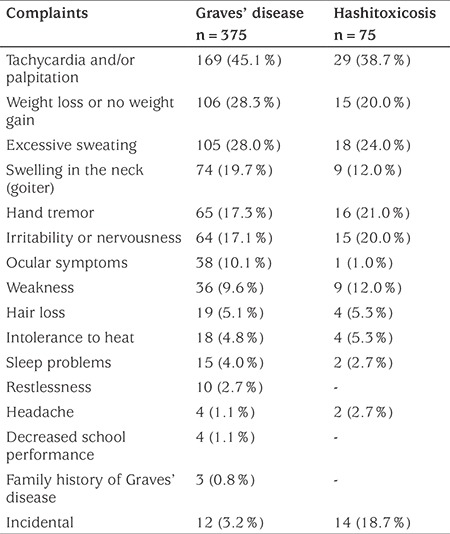
The most frequently reported presenting complaints among patients with Graves’ disease and hashitoxicosis. Data are given as n (%)

**Table 2:**
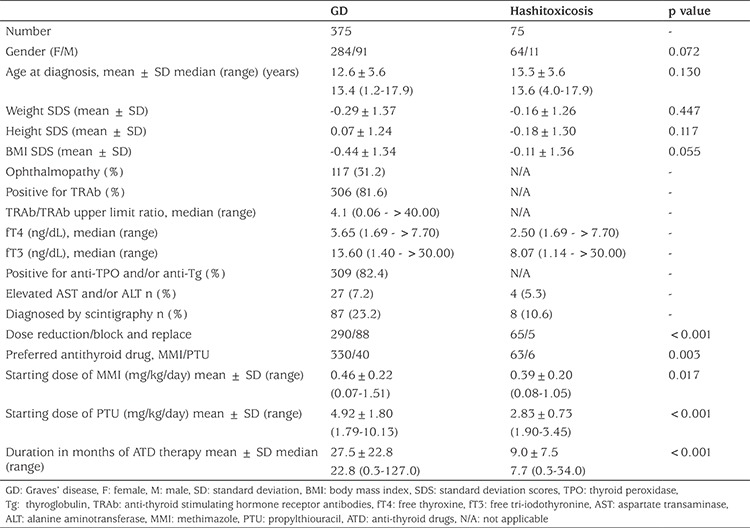
Clinical and biochemical features of patients with Graves’ disease and hashitoxicosis

**Table 3:**
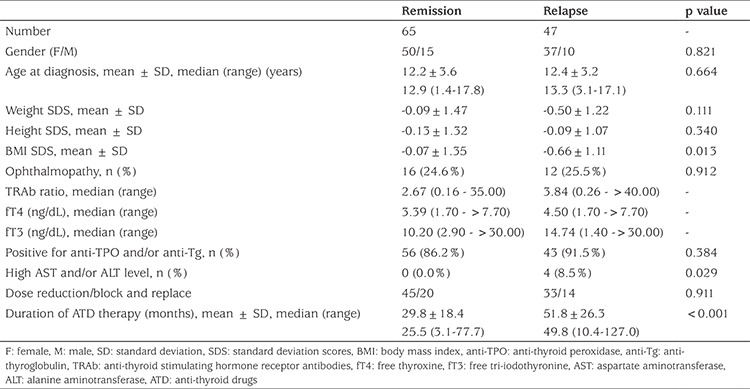
Clinical and biochemical features of patients with Graves’ disease who stopped anti-thyroid drug treatment for possible remission resulting in achieved remission or relapse

**Table 4:**
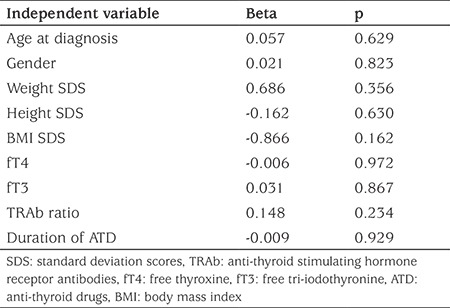
Multiple regression analysis in the patient group in which anti-thyroid drugs therapy was stopped for possible remission, with occurring relapse as the dependent variable
